# Glucose and lipopolysaccharide differentially regulate fibroblast growth factor 21 in healthy male human volunteers – A prospective cross‐over trial

**DOI:** 10.1111/jcmm.17614

**Published:** 2022-11-22

**Authors:** Johannes Pohlhammer, Matthias Wolfgang Heinzl, Carmen Klammer, Roland Feldbauer, Klemens Rosenberger, Michael Resl, Thomas Wagner, Florian Obendorf, Margot Egger‐Salmhofer, Benjamin Dieplinger, Martin Clodi

**Affiliations:** ^1^ Department of Medicine Konventhospital Barmherzige Brueder Linz (St. John of God Hospital Linz) Linz Austria; ^2^ ICMR–Institute for Cardiovascular and Metabolic Research, JKU Linz Linz Austria; ^3^ Department of Internal Medicine Klinikum Rohrbach Rohrbach Austria; ^4^ Department of Laboratory Medicine Konventhospital Barmherzige Brueder Linz and Ordensklinikum Linz Barmherzige Schwestern Linz Austria

**Keywords:** fibroblast growth factor 21, human endotoxin model, lipopolysaccharide

## Abstract

Fibroblast growth factor 21 (FGF21) affects the regulation of metabolism. Additionally, anti‐inflammatory properties are attributed to FGF21, and studies in animals and humans show conflicting results. This study aimed to investigate how FGF21 is affected by glucose and lipopolysaccharide (LPS) in humans. Therefore, FGF21 was measured eight times at different time points within 48 h in this prospective cross‐over trial after glucose and LPS on two different study days. The study included ten healthy, non‐smoking male subjects aged 18–40. Repeated measures analysis of variance and paired *t*‐test as post hoc analysis were applied. The administration of glucose and LPS resulted in a significant difference in regulating FGF21 (*p* < 0.001). After glucose administration, FGF21 declined sharply at 360 min, with a subsequent steep increase that exceeded baseline levels. LPS induced a drop in FGF21 after 180 min, while the baseline concentrations were not reached. After 180 min and 24 h, a statistically significant difference was demonstrated after adjusting the Bonferroni–Holm method. So, our results support the hypothesis that glucose and LPS differentially affect the human expression of FGF21 over 48 h.

## 1 INTRODUCTION

Endogenous fibroblast growth factor 21 (FGF21) is a peptide hormone and belongs to the FGF superfamily. The expression of FGF21 is mainly regulated by the peroxisome proliferation‐activated receptor‐α (PPAR‐α)^1^, ^2^, ^3^ and the carbohydrate‐responsive‐element‐binding‐protein (ChREBP)^4^, ^5^, ^6^ and is synthesized by the liver, pancreas, heart, musculature and adipose tissue.^7^ FGF21 signals in an autocrine, paracrine and endocrine way^8^ and binds to the FGF receptors (FGFR) type 1–4.^9^, ^10^, ^11^ The affinity depends mainly on the β‐Klotho co‐receptor complex, which is only found in the liver, pancreas, brain and adipose tissue.^12^, ^13^, ^14^ Recent data suggest that FGF21 exerts different effects according to the metabolic status to restore homeostasis.^15^ Independent of adiponectin,^16^ FGF21 improves insulin sensitivity through reduced hepatic gluconeogenesis and increased glucose utilization via activating glucose‐6‐phosphatase (G‐6‐Pase), adenosine‐monophosphate‐activated‐protein‐kinase‐α1 (AMPKα1), carnitine‐palmitoyl‐transferase‐1α (CPT1α) and carnitine‐palmitoyl‐transferase‐1β (CPT1β) in the liver.^17^ In addition, FGF21, as induced primarily in the liver via ChREBP after oral carbohydrate intake, seems to dampen the craving for more sweetened nutrients through direct signalling in the hypothalamus of the central nervous system.^4^, ^18^ However, the majority of the data relates to animal models.

Therefore, it is unsurprising that sustained hyperglycaemia is associated with increased FGF21 concentrations. Exemplarily, type 2 diabetes mellitus,^19^, ^20^ early stages of diabetic nephropathy,^21^ metabolic syndrome and obesity^22^ induce elevated FGF21 levels in humans. The poor prognosis of these severe diseases appears most likely due to persistent insulin resistance despite upregulated FGF21.^7^, ^15^, ^20^

Furthermore, FGF21 suppresses oxidative stress and inflammation.^7^, ^15^ In a mouse model and HepG2 cells, FGF21 administration ameliorates alcohol‐induced liver injury by targeting the adenosine‐monophosphate (AMP)‐activated‐protein‐kinase (AMPK) pathway. Additionally, FGF21 suppresses the oxidative stress markers malondialdehyde (MDA) and glutathione peroxidase (GSH‐PX) and inhibits intracellular reactive oxygen species (ROS) production.^23^ FGF21 also seems to counteract oxidative stress in humans. In human umbilical vein endothelial cells (HUVECs), the application of FGF21 leads to an attenuation of H_2_O_2_‐induced cell damage. The administration of FGF21 mitigates H_2_O_2_‐mediated cell apoptosis by reducing the ratio of apoptosis‐promoting protein Bcl‐2‐associated X protein (Bax) and apoptosis‐inhibiting protein B‐cell lymphoma‐2 (Bcl‐2). In addition, the application of FGF21 results in diminished depletion of caspase‐3 and reduces the expression of apoptosis and oxidative stress‐promoting kinases p38 and c‐Jun‐N‐terminal‐kinase (JNK), both belonging to the mitogen‐activated‐protein‐kinase (MAPK) group.^24^ In line with this, elevated serum levels of FGF21 are reported in patients suffering from severe clinical syndromes with a negative correlation on mortality independently of hyperglycaemia.^25^, ^26^, ^27^, ^28^, ^29^ Interestingly, increased serum levels of FGF21 are also described in patients with cardiac cachexia.^30^ Conceivably, this might be caused by attenuated FGF21 signalling^7^, ^31^ or by anti‐inflammatory effects in the presence of ongoing inflammation.^32^, ^33^, ^34^

The administration of lipopolysaccharide (LPS), a compound of the cell wall of gram‐negative bacteria, to induce artificial endotoxemia is a well‐established and proven model to generate a reliable systemic immune response in mice, cell models and humans. Depending on the dose administered, probands develop an illness with fever, chills, myalgia and arthralgia. Despite the frequent use, no severe side effects have been reported in the literature, so a high level of subject safety can be assumed.^35^, ^36^

In summary, we hypothesize that FGF21 plays a crucial role during inflammatory processes in addition to its known effects on metabolism in humans. The present cross‐over study aimed to determine whether FGF21 is influenced by glucose and LPS and if there is a difference in the human regulation of FGF21. The results are expected to improve our comprehension of the role of FGF21 in humans.

## 2 METHODS

### 2.1 Study participants and design

The study was designed as a prospective, single‐blinded cross‐over trial. The recruitment phase lasted from December 2017 to June 2018. A total of ten subjects were included after the following inclusion criteria were met: men aged 18–40 years, no disease history, no tobacco consumption, brain natriuretic peptide (BNP) within normal range, fasting glucose and haemoglobin A1c (HbA1c) within normal range, normal renal function (serum creatinine of 1.3 mg/dL and/or creatinine clearance greater than 80 ml/min). The volunteers were excluded for the following reasons to ensure high probands safety: systolic blood pressure below 90 millimetres of mercury (mmHg), probands on any medication, history of anaphylaxis, pathological thrombophilia screening and volunteers who suffered from infectious disease. They underwent a pre‐screening check‐up consisting of a physical examination, routine blood testing with comprehensive thrombophilia screening including single factor analysis, routine urine analysis and an electrocardiogram. All participants provided written informed consent. The study protocol was approved by the ethics committee of the Medical University of Vienna and the local research ethics committee (Institutional Review Board of the St. John of God Hospital Linz).

The subjects received bacterial endotoxin (intravenous injection of 2 ng/kg National Reference Bacterial Endotoxin) over 5 min together with saline 0.9% (200 ml/h) on the first study day due to logistical reasons. After a washout period of at least 14 days to avoid interference, 60 grams of glucose were injected intravenously in three single doses of 20 grams each over 5 min with one‐hour intervals on the second study day. The volunteers were examined at 08:00 a.m. after an overnight fast. They were encouraged to avoid caffeinated drinks 24 h before and during the study day. The participants were allowed to drink non‐sparkling mineral water, and upon completion of the whole study day, they were allowed to eat. The participants remained supine and were monitored consistently (non‐invasive blood pressure, heart rate, electrocardiogram and temperature). Intravenous catheters (B‐Braun) were placed into a vein on both arms as sampling and administration lines.

### 2.2 Preparation of LPS

U.S. Investigational Drug Management provided Standard Reference Endotoxin (lot #94332B1) at the National Institutes of Health (NIH), Bethesda, Maryland. Following good manufacturing practice guidelines, the purified lipopolysaccharide (LPS) was gained from Escherichia coli 0113. 10,000 Endotoxin Units (1 μg) per ampoule were delivered as a white, sterile, lyophilized powder. Before administration, the endotoxin was dissolved in sterile water and arranged regarding the manufacturer's recommendations.

### 2.3 Blood withdrawal and laboratory measurements

Blood specimens were obtained repeatedly. The first blood sample was taken after insertion of the intravenous sampling line. Blood samples were taken after administration of LPS or glucose at 08:00 a.m. and after 30, 60, 120, 180, 360 min, 24, and 48 h. The specimens at 24 and 48 h were obtained at 08:00 a.m. after overnight fasting. Using VACUETTE polyethylene terephthalate glycol blood collection tubes (Greiner Bio‐One), lithium‐heparin and ethylenediaminetetraacetic acid (EDTA), anticoagulated blood was gained.

FGF21 was analysed with proximity extension assay (PEA) and quantitative polymerase chain reaction (qPCR) in EDTA plasma performed by Olink proteomics (Upsala, Sweden). With this technique, the underlying antigens are bound by specific antibody pairs with deoxyribonucleic acid (DNA) oligonucleotide tails attached. The appropriated tails hybridize, are extended by DNA polymerase and are read off by qPCR [38]. The plasma levels are indicated in normalized protein expression (NPX) values. NPX reflects an arbitrary unit on a log2‐scale.

C‐reactive protein (CRP) serum concentrations were determined with standard assays on an Architect c16000 analyser (Abbott Diagnostics). Interleukin‐6 (IL‐6) was analysed with a chemiluminescent microparticle immunoassay on a Cobas e411 (Roche Diagnostics).

### 2.4 Statistical methods

Repeated measures analysis of variance (RM‐anova) was performed for statistical analysis. The Greenhouse–Geisser correction was applied if no sphericity could be assumed according to Mauchly's test of sphericity. For post hoc analysis of the individual time points, the paired t‐test was performed. The *p*‐values were adjusted for multiple testing based on the Bonferroni–Holm method. A *p*‐value less than 0.05 was considered statistically significant. In order to correct for baseline differences between the study days, values were adjusted for the respective baseline value using a ratio to the baseline for statistical calculations regarding FGF21. Analyses were performed with IBM SPSS Statistics 27.

## 3 RESULTS

### 3.1 Proband characteristics

**FIGURE 1:**
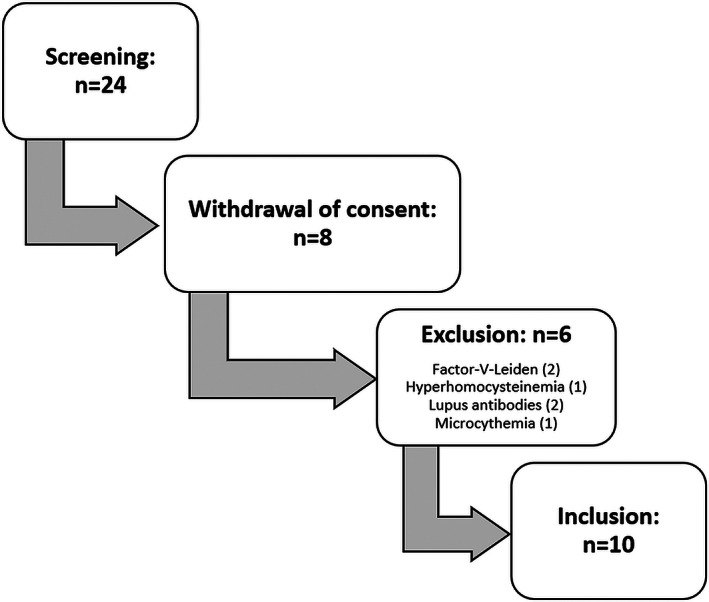
Flow chart illustrating the workflow of the study

In total, 24 healthy volunteers were screened, of which, eight withdrew their participation before enrolment, and six were excluded after screening according to the inclusion and exclusion criteria. For an overview, see Figure 1. A summary of the main baseline characteristics of the enrolled subjects is provided in Table 1. Subclinical inflammation was not detectable on admission as CRP and IL‐6 were within the normal range. Further details have been described previously.^37^

**TABLE 1:** Baseline characteristics of the included probands on the day of examination

	*Probands (n = 10)*
Age (years)	24.1 ± 3.7
Body mass index (kg/m^2^)	25.2 ± 1.6
FGF21 (NPX)	6.7 ± 1.6
C‐reactive protein (mg/dL)	0.11 ± 0.1
IL6 (pg/mL)	3.1 ± 2.2

Abbreviations: FGF21, Fibroblast Growth Factor 21; IL‐6, Interleukin‐6; kg/m^2^, kilogram per square metre; mg/dL, milligrams per decilitre; NPX, normalized protein expression value; pg/mL, picograms per millilitre; SD, mean ± standard deviation.

### 3.2 LPS induced inflammation

All but one of the recruited participants suffered from flu‐like symptoms with a maximum between 60 and 90 min after LPS infusion. The symptoms disappeared almost completely after 5 h. The corresponding data on CRP and IL‐6 after LPS have already been published.^37^

In contrast, glucose loading did not lead to any notable symptoms.

### 3.3 FGF21 kinetics

Following glucose administration, the plasma concentrations of FGF21 showed a concavely curved course with a slight peak at 180 min followed by a distinct drop at 360 min. Afterwards, the plasma values increased again and exceeded baseline values at 48 h.

The infusion of LPS led to an initially similar course with a sharp decrease at 180 min. After this drop, the baseline values remained distinctly below baseline until 48 h.

**FIGURE 2:**
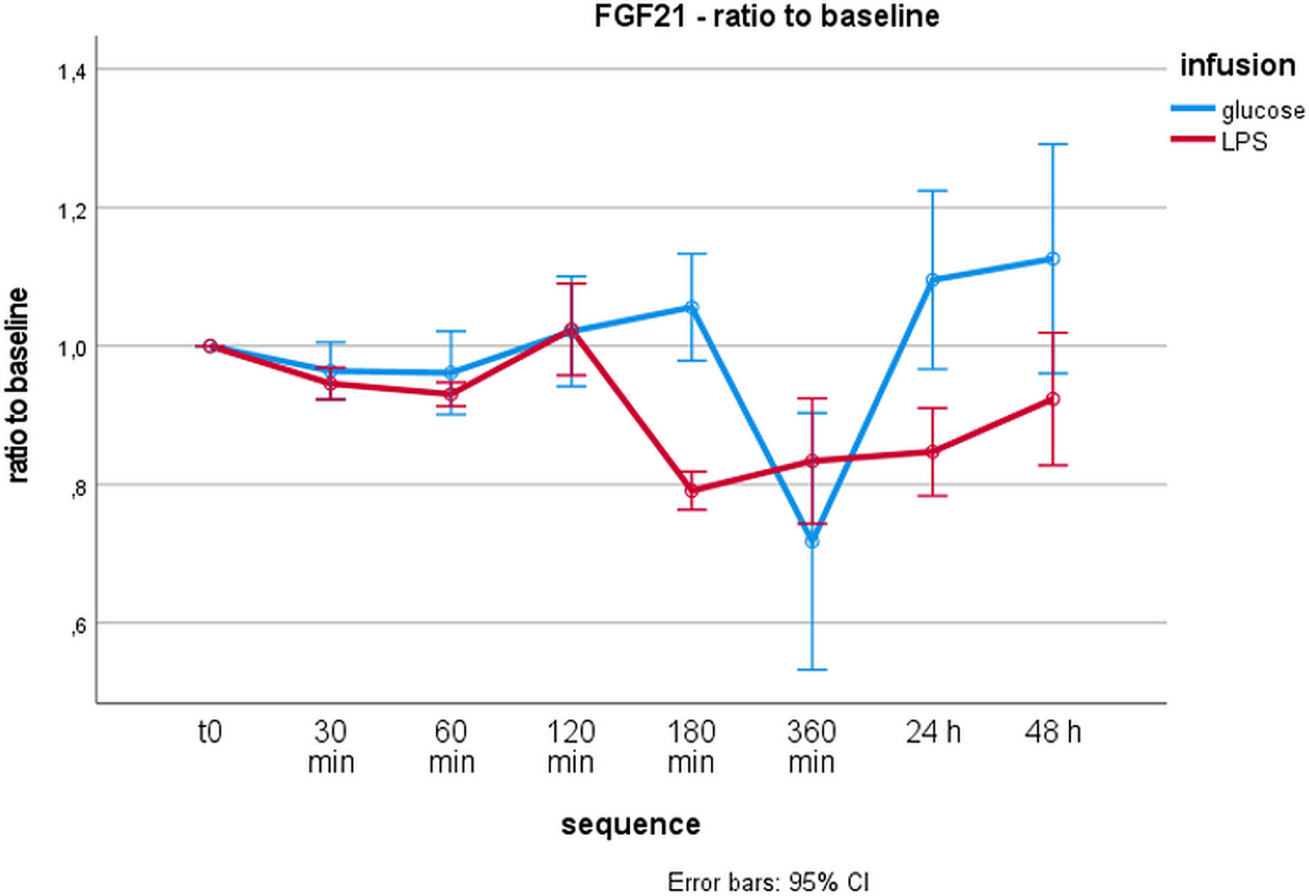
Repeated measures analysis of variance for Fibroblast Growth Factor 21 (FGF21). The difference in FGF21 plasma concentrations between glucose and lipopolysaccharide (LPS) administration was statistically significant (*p* < 0.001 using the Greenhouse–Geisser correction). The ratio of FGF21 values to baseline is shown on the ordinate, and the time points are shown on the abscissa. Error bars depict 95% confidence intervals

Both courses of FGF21 were statistically different (*p* < 0.001 as calculated by RM‐anova considering the Greenhouse–Geisser correction, outlined in Figure 2). The corresponding beta values (B), significance levels (*p*‐value) and 95% confidence intervals (CI) are provided in Table 2. In addition, RM‐anova was also performed with the original values (NPX) without correction for differences at the baseline. The corresponding results are listed in Table 3.

**TABLE 2:** RM‐anova was applied to investigate the difference in FGF21 plasma concentrations between LPS and glucose administration

Ratio to baseline and time point	B‐value	*p*‐value (entire model)	95% Confidence Interval (CI)	
			Lower Bound	Upper Bound
FGF21_LPS_t 0 min	1.000	<0.001	1.000	1.000
FGF21_glucose_t 0 min	1.000		1.000	1.000
FGF21_LPS_t 30 min	0.946		0.923	0.969
FGF21_glucose_t 30 min	0.964		0.923	1.005
FGF21_LPS_t 60 min	0.930		0.913	0.948
FGF21_glucose_t 60 min	0.961		0.901	1.021
FGF21_LPS_t 120 min	1.024		0.958	1.090
FGF21_glucose_t 120 min	1.021		0.942	1.101
FGF21_LPS_t 180 min	0.791		0.763	0.819
FGF21_glucose_t 180 min	1.056		0.979	1.133
FGF21_LPS_t 360 min	0.834		0.744	0.924
FGF21_glucose_t 360 min	0.718		0.532	0.903
FGF21_LPS_t 24 h	0.847		0.784	0.911
FGF21_glucose_t 24 h	1.096		0.966	1.225
FGF21_LPS_t 48 h	0.924		0.828	1.019
FGF21_glucose_t 48 h	1.126		0.961	1.292

*Note*: In order to correct for differences between the study days, values were adjusted for the respective baseline value by creating a ratio to baseline.

Abbreviations: <, less than; B‐value, Beta‐value; FGF21, Fibroblast Growth Factor 21; h, hour; LPS, lipopolysaccharide; min, minutes; RM‐anova, Repeated measures analysis of variance; t, time point.

**TABLE 3:** RM‐anova was applied to investigate the difference in FGF21 plasma concentrations between LPS and glucose administration

Original values (NPX) and time point	B‐value	*p*‐value (entire model)	95% Confidence Interval (CI)	
			Lower Bound	Upper Bound
FGF21_LPS_t 0 min	6.919	<0.001	5.723	8.116
FGF21_glucose_t 0 min	5.061		4.243	5.880
FGF21_LPS_t 30 min	6.605		5.373	7.837
FGF21_glucose_t 30 min	4.834		4.056	5.611
FGF21_LPS_t 60 min	6.456		5.353	7.558
FGF21_glucose_t 60 min	4.800		4.052	5.549
FGF21_LPS_t 120 min	7.023		6.079	7.968
FGF21_glucose_t 120 min	5.069		4.282	5.855
FGF21_LPS_t 180 min	5.492		4.479	6.506
FGF21_glucose_t 180 min	5.238		4.482	5.995
FGF21_LPS_t 360 min	5.726		4.622	6.831
FGF21_glucose_t 360 min	4.031		3.361	4.701
FGF21_LPS_t 24 h	5.938		4.538	7.338
FGF21_glucose_t 24 h	5.674		4.463	6.885
FGF21_LPS_t 48 h	6.462		4.742	8.182
FGF21_glucose_t 48 h	5.540		4.935	6.146

*Note*: The calculation was performed with the original values (NPX) without considering any differences between the study days.

Abbreviations: <, less than; B‐value, Beta‐value; FGF21, Fibroblast Growth Factor 21; h, hour; LPS, lipopolysaccharide; min, minutes; NPX, normalized protein expression; RM‐anova, Repeated measures analysis of variance; t, time point.

Post hoc analysis of the individual time points revealed that FGF21 was significantly differentially regulated after 180 min (*p* < 0.001) and after 24 h (<0.001), considering the Bonferroni–Holm method. The corresponding results of all time points are provided in Table 4.

**TABLE 4:** Paired t‐test was applied to investigate the difference in FGF21 plasma concentrations between LPS and glucose administration at each time point

Ratio to baseline and time point	Mean	*p*‐value (Bonferroni–Holm)	*p*‐value (two‐sided)	95% Confidence Interval (CI)	
				Lower Bound	Upper Bound
FGF21_t 30 min	−0.019	>0.999	0.433	−0.070	0.033
FGF21_t 60 min	−0.032	>0.999	0.341	−0.102	0.039
FGF21_t 120 min	0.003	>0.999	0.943	−0.085	0.091
FGF21_t 180 min	−0.266	<0.001	<0.001	−0.340	−0.191
FGF21_t 360 min	0.116	0.880	0.220	−0.083	0.314
FGF21_t 24 h	−0.249	<0.001	<0.001	−0.354	−0.144
FGF21_t 48 h	−0.203	0.120	0.024	−0.372	−0.034

*Note*: In order to correct for differences between the study days, values were adjusted for the respective baseline value by creating a ratio to baseline. Therefore, the analysis was omitted at time 0 min, which corresponds to the baseline. In order to consider multiple testing, the p‐value is additionally adjusted based on the Bonferroni–Holm method.

Abbreviations: <, less than; FGF21, Fibroblast Growth Factor 21; h, hour; LPS, lipopolysaccharide; min, minutes; NPX, normalized protein expression; t, time point.

## 4 DISCUSSION

The study at hand aimed to investigate the response of FGF21 in humans after LPS and glucose administration. Over 48 h, our data suggest that FGF21 is regulated distinctly by inflammation and after glucose load in humans. A remarkable observation of our study is that FGF21 is suppressed by both LPS and glucose but at different time points. Additionally, in contrast to glucose administration, FGF21 increases gradually in the further course after LPS loading. It can be concluded that the timing of the analysis of FGF21 has a decisive impact on its serum levels.

The biology and mode of action of FGF21 are genuinely complicated and still insufficiently understood. Another complicating factor is that human regulation different from animal models.^7^, ^8^ This aspect was one of the main reasons for investigating FGF21 regulation in humans in this study.

In our trial, administration of 60 grams of glucose in three single doses of 20 grams each led to a modest increase of FGF21 over 48 h, including a sharp and not persistent decline at 360 min. After oral ingestion of 75 grams of glucose, a similar observation was made in previous studies but with a shorter observation period.^38^, ^39^ Interestingly, these studies recorded the drop of FGF21 serum concentrations much earlier, most likely due to higher glucose intake. Fold changes in FGF21 were significantly negatively correlated with fold changes in glucose, insulin and C‐peptide in healthy subjects.^38^ Chavez A.O. et al. reported increased FGF21 plasma concentrations in insulin‐resistant conditions such as type 2 diabetes mellitus, impaired glucose tolerance and obesity compared to human subjects with normal glucose tolerance. Additionally, FGF21 levels were directly correlated with peripheral and hepatic insulin resistance.^40^ As FGF21 directly suppresses lipolysis in adipose tissue through decreased expression of perilipin (PLIN)^41^ and inhibits hepatic gluconeogenesis^17^ by affecting glucose‐6‐phosphatase (G‐6‐Pase), carnitine‐palmitoyl‐transferase‐1α and carnitine‐palmitoyl‐transferase‐1β (CPT1α and CPT1β), adenosine‐monophosphate‐activated‐protein‐kinase‐α1 (AMPKα1), plasma levels of FGF21 are most likely increased in a compensatory manner.^40^ Moreover, recent studies suggest that adiponectin, considered an insulin‐sensitizing hormone, appears to induce FGF21 in the liver and is unnecessary for its effects on target tissues.^16^, ^42^

Crucial for the effect of FGF21 is the co‐expression of β‐Klotho on target tissues.^12^, ^13^ It was reported that β‐Klotho protein levels in visceral and subcutaneous fat were significantly suppressed in obese patients compared to lean subjects, whereas the opposite was found in the liver.^43^ FGF21 was directly correlated with insulin resistance, although statistical significance was lost after adjustment for body mass index (BMI). An inverse correlation with insulin resistance was found for fibroblast growth factor 19 (FGF19) in this study, which also showed a dependence on body mass index (BMI).^43^ In mouse models, similar metabolic effects of FGF19 were suspected with FGF21.^44^, ^45^ Despite opposite changes in FGF19 and FGF21 in obese and insulin‐resistant patients,^46^ both seem to exhibit widely overlapping metabolic effects.^43^

A further important role of FGF21 is its impact on nutritional intake. Sugar consumption inevitably leads to increased expression of FGF21 by carbohydrate‐responsive‐element‐binding‐protein (ChREBP) in the liver of both mice and humans.^4^, ^18^, ^39^, ^47^ The craving for sweets seems to be curbed through direct signalling of FGF21 in the hypothalamus of the central nervous system,^4^, ^18^, ^48^ although the intake of lipids and protein is not affected.^4^, ^49^ Interestingly, fasting differentially influences the expression of FGF21. It is known that only fasting for at least 72 h leads to lower FGF21 serum concentrations, whereas a ketogenic diet or acute overnight fasting has no effect on FGF21 in humans.^7^, ^8^ FGF21 can rapidly and substantially improve insulin sensitivity by direct signalling to adipose tissue as reported in a mouse model with β‐Klotho‐adipose‐tissue knockout (KLB AdipoKO) mice^16^ and by reduced hepatic gluconeogenesis and by increased glucose consumption via activating G‐6‐Pase, AMPKα1, CPT1α, CPT1β. Therefore, we hypothesize that the expression of FGF21 after glucose loading for 48 h in this study is due to an acute and unsustainable change in insulin sensitivity. However, the involvement of other adipostatic hormones in regulating FGF21 cannot be safely excluded.^50^

Besides the notable impact on lipid and glucose metabolism, FGF21 has potential anti‐inflammatory effects, possibly explaining the increase in chronic inflammation.^7^, ^23^, ^24^, ^32^, ^33^, ^34^, ^51^, ^52^ To the best of our knowledge, Lauritzen et al. were the first to investigate the acute inflammatory response of FGF21 in human subjects over 4 h.^53^ Only bolus administration of LPS resulted in a statistically significant difference compared with saline resulting in a late decrease of FGF21 after 240 min. In our study, the decrease was detected between 120 and 180 min, most likely due to the higher dose of LPS (1 ng/kg vs. 2 ng/kg). After that, FGF21 increases only gradually, and the baseline values are not reached until 48 h.

In an animal model with male rats, high‐dose LPS (5 mg/kg) was applied and induced severe hepatic injury.^54^ The injection of LPS induced hepatic production of FGF21 and increased serum concentrations. Administration of metformin, an anti‐diabetic drug with anti‐inflammatory effects through the inhibition of nuclear factor kappa B (NF‐κB) via the adenosine‐monophosphate (AMP)‐activated‐protein‐kinase (AMPK) pathway^55^, ^56^ and the induction of interleukin‐4 and interleukin‐10,^57^ markedly reduced inflammation. Furthermore, metformin appears to stimulate hepatic FGF21 production.^54^ The authors suggest that FGF21 and metformin together have a liver‐protective impact through anti‐inflammatory effects.^54^

Moreover, it was demonstrated in male mice that FGF21 directly influences the polarization of macrophages. LPS induces the differentiation into proinflammatory M1 macrophages, whereas FGF21 causes a reduction of M1 markers towards the development of anti‐inflammatory M2 macrophages. Again, FGF21 can attenuate inflammation‐related harm via the AMPK/NF‐κB signalling pathway.^58^

As inflammation induces multiple endocrine modifications and puts the human body under stress,^59^ we assume that FGF21 is differently regulated in humans during acute inflammation. While FGF21 is upregulated in chronic inflammation to provide anti‐inflammatory effects, for example, by activating the AMPK pathway or inhibiting intracellular ROS,^23^ it appears not to be the same in acute inflammation in humans. Considering our data most widely confirm those of Lauritzen et al., we suggest that the inhibition of FGF21 is intended to achieve a significant human acute immune response. Our data do not support the results of a leptin‐deficient mouse model, where FGF21 is considered to fulfil the function of a positive acute‐phase protein.^32^

In summary, our results indicate different pathways in regulation after glucose and LPS in humans, and plasma concentrations of FGF21 are crucially affected by the time of lab analysis.

### 4.1 Strengths and limitations of the study

We consider it a strength that we performed our study on humans. We only included male volunteers to exclude hormonal fluctuations as far as possible. Whether our results can also be extrapolated to women has to be investigated. Another strength is the close‐meshed blood sampling to show acute changes in FGF21. Due to the nature of this cross‐over study, it is not easy to draw a causal conclusion. No pre‐emptive sample size calculation was performed based on the unknown effect size. However, although the number of participants was small, it was adequate to demonstrate a statistically significant result.

## 5 CONCLUSION

Our results demonstrate that FGF 21 is differentially regulated after LPS administration and glucose loading in ten healthy male volunteers over 48 h. Further studies are required to determine the underlying mechanisms to improve the comprehension of the physiology of FGF21 in humans.

## AUTHOR CONTRIBUTIONS

**Johannes Pohlhammer:** Writing – original draft (lead). **Matthias Wolfgang Heinzl:** Writing – review and editing (equal). **Carmen Klammer:** Writing – review and editing (equal). **Roland Feldbauer:** Writing – review and editing (equal). **Klemens Rosenberger:** Writing – review and editing (equal). **Michael Resl:** Writing – review and editing (equal). **Thomas Wagner:** Writing – review and editing (equal). **Florian Obendorf:** Writing – review and editing (equal). **Margot Egger‐Salmhofer:** Writing – review and editing (equal). **Benjamin Dieplinger:** Writing – review and editing (equal). **Martin Clodi:** Writing – review and editing (lead).

## CONFLICT OF INTEREST

The authors state that they have no conflicts of interest.

## Data Availability

The data that support the findings of this study are available from the corresponding author upon reasonable request.
